# From classrooms to real-world contexts: enhancing vaccine education through open schooling

**DOI:** 10.3389/fpubh.2025.1520395

**Published:** 2025-01-29

**Authors:** Hannah Kwella, Jana Schilbert, Amélie Tessartz, Annette Scheersoi

**Affiliations:** University of Bonn, Bonn, Germany

**Keywords:** vaccine education, open schooling, socio-scientific issue, real-world contexts, ethical aspects

## Abstract

The topic of vaccination has been a highly debated issue for many years, whether related to measles, HPV, or the recent COVID-19 pandemic. It necessitates deeper exploration, particularly in school biology classes where it is often superficially covered, with ethical considerations rarely addressed. To enable students to engage in an in-depth examination of this complex socio-scientific issue and to enhance their argumentation and decision-making skills, a vaccine educational project was implemented based on the concept of open schooling, where schools collaborate with various societal institutions. Over a three-day interdisciplinary program, secondary school students worked with scientists from diverse fields, including immunobiology, medicine, and ethics, across different career levels, providing varied perspectives. Students actively engaged in real-world learning contexts with authentic problems, fostering individual reflection. A qualitative study, which involved observations and interviews with students, scientists, and teachers, highlighted key success factors in developing student interest and engagement in the topic of vaccination: learner-centered design, interaction with experts, exposure to diverse professional environments, active science learning, and the integration of ethical aspects. This approach promoted not only student engagement with the complex subject matter but also critical thinking and argumentation, contributing to informed decision-making and public health awareness.

## 1 Introduction

Vaccination is one of humanity’s greatest public health achievements, widely recognized for its effectiveness in preventing major diseases such as diphtheria, tetanus, polio, and influenza (37). Despite this, vaccine hesitancy and declining immunization rates continue to rank among the top ten global health challenges, underscoring the urgent need for better vaccine education.

Recent studies emphasize the positive impact of comprehensive educational initiatives focused on vaccination on countering vaccination hesitancy and immunization rates (1). Although these efforts can be carried out in various contexts, Schott et al. (2) found that school-based education is particularly effective in promoting HPV vaccination, as it reaches a wide audience of students and their families. Another advantage of the school setting is that students spend a significant amount of time there, and instruction is generally delivered by highly trained educators (3).

In school curricula, however, vaccination is often only treated as a secondary topic, usually connected to broader subjects like the immune system or genetics (3, 4). Most student tasks focus narrowly on scientific content, neglecting emotional and socio-cultural dimensions, which raises concerns about the adequacy of this approach in modern science education (5). When addressing contentious topics like vaccination, educational efforts must move beyond simply presenting facts. Students need to be equipped with the skills to critically assess and contextualize the information they receive (3, 6). To provide a comprehensive understanding and support public health, the scientific aspects of vaccination should be taught alongside its ethical and societal implications (3), such as personal autonomy, public health, and vaccine hesitancy.

The complexity of the vaccination topic necessitates that learners engage deeply with the material to fully understand and evaluate it. Cultivating students’ interest in the content is essential for ensuring such engagement. Interest is a key motivational factor in (life-long) learning, as it fosters persistence, and voluntary engagement with learning materials, thereby making the learning experience more meaningful and effective (7, 8). However, there is a lack of research on how to foster student interest in vaccination and develop effective instructional strategies (1, 3, 9).

The open schooling approach, which emphasizes real-world contexts, interdisciplinary learning, and student-centered pedagogy, may serve as an effective framework for promoting the students’ interest and engagement and thoroughly exploring the topic of vaccination while combining scientific content with ethical and social discussions. Therefore, this study investigates an open schooling program focused on vaccination to identifying key factors that foster student interest and engagement, with the goal of improving contemporary vaccine education that integrates both scientific learning and its social implications.

Our study has been conducted as part of the international open schooling project Multipliers,^1^ within which students engaged with various socio-scientific issues and current challenges. In our subproject, the focus was on exploring the topic of vaccination. The study involved secondary school students participating in a three-day interdisciplinary vaccine education program alongside scientists from fields such as immunobiology, medicine, and ethics. Throughout the program, students actively engaged in discussion and argumentation processes. Afterwards, interviews were conducted with students, scientists, and teachers to explore their experiences and identify interest-enhancing factors.

The insights gathered from these interviews informed the development of recommendations for designing innovative educational projects in real-world contexts, aimed at promoting contemporary science education and improving vaccination education.

## 2 Background

Vaccination is a complex topic, not only due to its foundational biological principles but also because of its significant societal relevance and the diverse, often controversial attitudes associated with it. To ensure that students engage deeply and comprehensively with this topic, it is essential to spark and maintain their interest, which in turn promotes knowledge acquisition and argumentation skills. Research indicates that students’ interest and engagement with (classroom) topics are enhanced when they have the opportunity to work hands-on—ideally in authentic contexts that differ from the often theory-heavy school curriculum—when they recognize the topic’s relevance to their own lives or to society, and when they can have unique and novel learning experiences [e.g., (10–14)]. Additionally, approaches tailored to students’ needs are crucial for motivation and learning, ensuring they are neither under- nor over-challenged, while also taking their subject-specific knowledge and methodological skills into account (15).

A pedagogical approach that encompasses these interest-promoting aspects is the open schooling approach, which is gaining increasing importance in science education. The European Commission (16) emphasizes open schooling as a transformative approach that positions schools as collaborative agents of community well-being. This model involves diverse stakeholders—including professionals from enterprises, civil society, and the broader community—who bring real-life projects into the classroom. Open schooling employs project-based, inquiry-driven methodologies, encouraging schools to work with local stakeholders to address real-world problems and enhance community well-being (17).

By focusing on inclusive, community-focused projects, open schooling aims to address local needs while fostering global awareness and sustainability (18–20). These initiatives align educational practices with the United Nations’ Agenda 2030 goals, making schools active contributors to sustainable development (21).

Within this framework, learning extends well beyond the classroom, allowing students to gain firsthand exposure to scientific practices in diverse environments, such as science labs, museums, science centers, and community projects. By connecting science to real-life applications, open schooling encourages students to view science as a meaningful and impactful field. This practical exposure enhances students’ engagement with science and may positively influence their science-based career aspirations (22). Given its potential impact, the concept of open schooling and its role in shaping contemporary science education are now subjects of intensive research (e.g., (23)).

Building on this background, we investigate whether an open schooling program on vaccination impacts secondary school students’ interest and engagement with the topic and which factors contribute to this.

## 3 Materials and methods

To describe the methods of this study in detail, we will first present the planning and implementation of the teaching module, followed by a detailed explanation of the data collection and analysis methods.

Our vaccine education program was implemented using a systematic approach, beginning with preliminary research, followed by a collaborative planning phase, and culminating in a three-day educational module.

### 3.1 Preliminary research

Before the planning phase, we conducted desk research on existing vaccination educational materials. Additionally, we carried out an interview study with secondary school students, experienced teachers, and vaccination experts to identify factors that foster student engagement and interest in immunobiology and vaccines. The findings from these studies, published by Schlopsna and Scheersoi (9), were instrumental in shaping the lesson plan, ensuring that the program addressed key pedagogical needs and interests.

### 3.2 Collaborative planning

The planning phase centered on collaboration between teachers and experts from various fields. Although students were not directly involved in this phase, their prior knowledge, methodological skills, and areas of interest were carefully considered based on data from our preliminary research (9). This ensured that the educational module remained relevant to students’ lives and aligned with the curriculum.

In the planning of the educational program, we engaged experts from a range of disciplines, including biology, medicine, and ethics, as well as senior scientists and young researchers (PhD students and postdoctoral researchers) working on vaccine-related topics. The early involvement of these experts contributed to ensuring that the scientific content was accurate and communicated effectively to younger audiences. To better equip the experts for working with secondary school students, we organized a workshop where researchers from biology education discussed the experts’ previous experiences with students and their methodological approaches. This allowed the identification of potential challenges and provided advice on suitable teaching methods for younger learners.

The planning phase was conducted through a combination of two on-site meetings and several video calls. These sessions allowed project partners to discuss key vaccination topics, explore how to best leverage scientific expertise in the educational setting, and decide on the virus to use as a central example for explaining vaccination concepts.

Table 1 summarizes the participants and their roles in the collaborative planning phase.

**Table 1:** Experts involved and their role in the planning phase.

Science Research Institution (University)	Researchers specializing in biomedicine and immunology contributed scientific knowledge, helping to ensure the accuracy and depth of the content on vaccines and immunology.
Non-profit Association for Vaccination Education	Medical students from a local university brought practical medical perspectives and experience from previous programs with students including interactive sessions.
Ethics Institution (University)	Researchers in ethics and philosophy provided guidance on the ethical implications of vaccination, helping to incorporate discussions on ethical decision-making into the program.
Secondary School	Two experienced teachers played a key role in aligning the program with educational curricula and ensuring the material was accessible to students.
Education Research (University)	Researchers in biology education provided pedagogical expertise, supporting the development of teaching methods and ensuring that the program effectively engaged students in scientific inquiry.

The primary objectives of these meetings were to establish an open and transparent communication structure and to foster a collaborative environment among all participants from the start.

### 3.3 Three-day educational module

The educational module was conducted at a STEM-focused secondary school over three consecutive afternoons (see Table 2). Participants included 25 students aged 16 to 17 years, all enrolled in an advanced biology course. Participation was voluntary, and all activities were held outside of school premises.

**Table 2:** Overview of the educational module.

	Theme	Goal	Short Description	Experts involved
Day 1	Content knowledge: Immune system and vaccination	Provide students with foundational knowledge about the immune system and the principles of vaccination, while fostering engagement through interactive activities	PPP presentation and game-like activities in a university lecture hall	Senior scientist in immunology; medical students
Day 2	Practical laboratory experience	Offer students hands-on experience in a research laboratory setting, allowing them to apply theoretical knowledge to practical experiments, enhance their technical skills, and explore various areas of biomedical research	Visit to university’s research laboratories including hands-on investigations; Q&A session with early career researchers	Researchers in biomedicine and immunology (different career levels)
Day 3	Ethical considerations in vaccination	Encourage critical thinking and ethical reasoning	Roleplay discussion related to compulsory COVID-19 vaccination at a university’s ethics institution	PhD student in ethics; medical student

The educational module spanned three days, each dedicated to different aspects of immunology and ethics:

On the first day, students focused on immune system and vaccination facts at the local university’s lecture hall. A senior immunology scientist delivered a lecture using a PowerPoint presentation, while medical students facilitated interactive, game-like activities to engage participants.

The second day provided students with practical laboratory experience at university research center laboratories. Young biomedical and immunology researchers guided students through hands-on investigations, including plasmid transfection and gel electrophoresis. Students could choose which laboratory they wished to visit based on short films recorded in advance by the experts, allowing them to explore areas aligned with their individual interests. After the laboratory activities, a question-and-answer session offered students further insights and the opportunity to interact with the researchers.

On the final day, held at a university’s ethics institution, students explored ethical considerations surrounding vaccination. Prior to this session, students were provided with preparatory materials and web access to familiarize themselves with the topic. A PhD student in ethics facilitated a fictional citizens’ council meeting, simulating a scenario in which participants discussed and made recommendations on public policy issues related to compulsory COVID-19 vaccination. To ensure the accuracy of the factual knowledge presented, a medical student was also present to assist. This exercise aimed to enhance students’ critical thinking and argumentation skills.

### 3.4 Data collection

Throughout the intervention, data were collected to identify factors that enhanced student interest and engagement within the educational setting. Our study aimed to conduct an in-depth case study of a specific open schooling program to explore its impact on student learning. To achieve these objectives, qualitative research methodologies were employed, as they are particularly well-suited for exploring new research areas and generating context-specific insights [e.g., (24)]. The primary aim of our study was not to examine behavioral patterns or produce statistical generalizations. Instead, we focused on generating rich, detailed insights into how students engage with and learn about vaccination in a real-world classroom setting. Aligned with this purpose, our research emphasizes qualitative inquiry, focusing on context-specific meanings and reflexive processes that shape the learning experience.

Interviews were conducted with students, experts, and the teachers involved (see Tables 3, 4). While the teachers and experts were interviewed individually, the students participated in small group interviews (3–4 students per group). This format was chosen to reduce anxiety and foster a conversational atmosphere, encouraging more authentic responses, as students could exchange ideas and express their preferences and interests (25). To minimize the risk of influencing respondents’ answer patterns through predefined response options, a standardized survey was not utilized. Instead, open-ended semi-structured interviews were conducted, maintaining flexibility, as additional questions and topics can be addressed spontaneously based on the course of the interview (25). This flexibility is particularly valuable in qualitative research, where unexpected insights can emerge during the interview process.

**Table 3:** Data collection, interview.

Participants (*N* = 18)	Teachers (2), students (12), experts: science (3), ethics (1)
Timing of data collection	Data was collected during and after the educational module
Data collection method	Group interviews with students (3–4 students per group); individual interviews with the teacher and the experts

**Table 4:** Interview participants.

Expert #1: Researcher in immunology	Science Research Institution (University)
Expert #2: Medical student Expert #3: Medical student	Non-profit Association for Vaccination Education
Expert #4: PhD student in ethics	Ethics Institute (University)
Teacher #1 Teacher #2	Secondary School
Student #1–12	16–17 years, Secondary school/advanced biology course

Purposive sampling was employed to select participants who were willing to provide insights into the research topic. This targeted sampling approach allowed us to closely examine the program, focusing on students’ individual experiences and perspectives. Detailed information regarding the study’s purpose, the significance of their participation, and the potential impact of their responses was communicated to both the students and their parents. Parental consent was obtained for all participants, and confidentiality was maintained throughout the process. To enhance credibility and dependability, member checking was utilized by sharing key findings with participants for validation.

During data collection, triangulation was employed to strengthen the study’s validity. First, triangulation by data source (26) was used by revisiting the same themes across interviews with students, teachers, and experts. This approach facilitated comparison of participants’ statements, enhancing the accuracy of interpreting recurring responses. For example, teachers and experts were asked to identify the topics and activities they observed as most engaging for students. Second, triangulation by method (26) was implemented by combining interviews with participant observations conducted throughout the three-day program. This comprehensive data collection approach aimed to provide a holistic understanding of the program’s effectiveness in fostering students’ interest and engagement with the complex topic of vaccination.

Observations were initially conducted using minimal standardization (27), with the aim of noting instances where students demonstrated interest and engagement, such as focused work, lively idea exchanges, or enjoyment of activities. Observational notes were recorded in bullet points during the program and elaborated in detail immediately after fieldwork, including external circumstances, notable events, and key participant statements.

### 3.5 Data analysis

The interview data were audio recorded and subsequently transcribed into text files (28). Both the data from interviews and observations were then systematically analyzed using qualitative content analysis: Statements from the transcripts were condensed into thematic units and organized into categories, following the content-structuring approach outlined by Kuckartz (29). This method is well-suited to capturing nuanced, textual information because it allows researchers to both apply predefined categories and develop new ones inductively during the analysis process. In our case, the initial category system was informed by our research question and the study’s background (characteristics of open schooling programs and interest-promoting factors). As we engaged with the data, we refined this system by adding inductive categories to account for emerging themes that were not anticipated in the original framework (e.g., benefits of involving experts at different career levels). This systematic approach allowed us to explore the conditions, processes, and experiences that shaped students’ engagement with the topic of vaccination in a nuanced and context-sensitive manner.

To ensure transparency, the results section presents original interview quotes alongside their corresponding categorizations and interpretations (Tables 5–9). This approach allows readers to critically assess the data analysis and ensures a transparent, traceable link between the raw data and the conclusions drawn.

**Table 5:** Selection of quotes from different groups of participants describing ‘Collaborative planning’ as a key success factor.

Theme	Subtheme	Example quotes
Collaborative planning	Collaboration provides valuable feedback	*“The day (with the students) itself was the highlight; but the planning beforehand was also quite interesting because it was different from usual. Sure, we often do school presentations, but this time we got additional input from a different perspective. (…) We received direct input from the school, or from people who might be more scientifically engaged than we are and who see things a bit differently. That’s something we usually do not experience.”* (Expert #2, medical student)
		*“None of us have any didactic or pedagogical training, and we do it based on feeling, and it’s very valuable to receive such feedback from that perspective.”* (Expert #3, medical student)
		*“Your suggestion to involve the teacher was very helpful (…). I had not even considered that they need a role at that moment. And that you can utilize the potential that is there.”* (Expert #4, ethics researcher)
		*“I had sent you the presentation beforehand, and you had given me feedback on that, which I also incorporated. So that was actually a great support, especially from people outside the subject, when there were quite a few technical terms in it, to simplify that as well.”* (Expert #1, researcher in immunology)
	Collaborative planning to enhance the expert-student alignment	*“It was a unique situation because, normally, you do not have experts readily available to discuss how to proceed together. Typically, you prepare your lessons and then seek out experts who might fit afterward. […] Because of this, the experts were able to adjust very well to our student. I usually encounter the problem that when set up appointments with experts, they do not really understand where the students are in their learning […]. I had the feeling that the experts were closer to the students because they already knew what to expect.”* (Teacher #2)
	Collaborative planning as a basis for continuing collaboration	*“I think it’s super important that we had personal exchange because I believe that if you do not meet directly, no long-term collaboration arises.* “(Expert #2, medical student)

**Table 6:** Selection of quotes from different groups of participants describing ‘Interaction with experts’ as a key success factor.

Theme	Subtheme	Example quotes
Interaction with experts	Difference to regular classes and interaction with teachers	*“Knowing that someone is coming who’s an expert in that field, definitely made a difference, compared to if I had just said we’ll do it in class, grade it, and that’s it. It certainly had a positive effect.”* (Teacher #1)
	Insights into professional routines and real-world activities	*“That gives a look into the field, into the profession, and in that sense, I could certainly imagine that it could be a guiding experience for some.”* (Teacher #1)
		*“Even afterwards […] after the lesson was officially over, they took time to show us the lab, which I found very helpful, and we could also ask people there what they were working on. I found that very helpful.”* (Student #2)
		*“And now, we were behind the scenes because we talked to the people and did not just see them from the outside but had direct contact with them.”* (Student #3)
		*“[Through my family] I already had a bit of contact [with scientists], but the whole biology and chemistry aspect was completely new, and I really liked that. I always thought, ‘Yeah, you just do something in the lab,’ and now I actually know what they do and what their daily routine is like. I thought that was really cool.”* (Student #8)
		*“I also appreciated how they interacted with us, and how they kept assuring us: ‘You’re really doing something related to science here, something we have done before or do in our daily work.’ That […] took away the sense of distance.”* (Student #7)
	Accessibility of experts	*“The scientists did not expect us to do everything perfectly; they were like: Yeah, it’s okay if a mistake happens, and they were very relaxed about it. I found that it had a calming effect, that you did not have the stress of ‘I have to get everything right’.”* (Student #11)
		*“I really liked that because you always think, ‘Oh, that’s so reserved,’ but there it felt really normal. You could talk, ask anything, and it was really nice.”* (Student #8)
	Benefits of involving different career levels	*“I could have sent a PhD student or a post-doc, and they would have done just as well. But you likely asked for professors to attend because it might make a bigger impression.”* (Expert #1, researcher in immunology)
		*“Personally, I thought it was very cool to see very young people […]. You could really tell they were still passionate about their topic. Because I think, the older you get, the more accustomed you become, meaning you no longer have quite the enthusiasm that you can have when you are just starting out. […]”* (Student #12)
		*“Especially the students, they were not quite experts themselves yet, you had the feeling they were still somewhat on the same level. I mean, the others had more knowledge, so you knew you could always ask them, but I always felt it was very good to work with real experts from biology and then also with students.”* (Student #4)
		*I also thought the [medical students’] team was great, especially because they were so young—they were like a bridge to everything else. I was really nervous, but then I saw them, and they were so nice, which is why I think they were really important.”* (Student #8)

**Table 7:** Selection of quotes from different groups of participants describing ‘Exposure to authentic workplaces’ as a key success factor.

Theme	Subtheme	Example quotes
Exposure to authentic workplaces	Leaving the classroom	*“I think, for example, the setting, that it took place at the university, was totally motivating for the students. […]* *I think it gives the students the feeling that they are now engaging with science, which might spark a bit more interest or motivation.”* (Expert #3, medical student)
		*“Having a practical phase outside of school perhaps, […] that’s of course another story entirely. I think that could generate significantly more momentum and engagement, simply because you go out, see something different. The visits at school are nice, and of course, it’s great when something external comes in, but seeing a lab and working there, entering an institute–that has a completely different motivational effect.*” (Teacher #1)
	Insights into research environments	*You could really see how they work there and how they analyze things. That’s why the second day was my favorite.”* (Student #4)
		*“You learn a lot of new things, and you also gain experience in the lab […].”* (Student #4)
		*“I would actually recommend it because you not only gain the knowledge but also get a glimpse of everyday life in a lab.”* (Student #6)
		*“[…] The places we saw–we were in the lecture hall center at one point, and I actually thought it was pretty cool. So, in a way, it inspired me a bit, like, ‘Okay, so this is what it looks like at a university’”* (Student #9)
	Uniqueness of such insights	*“My highlight was the lab. You do not see that every day because labs are usually closed off to the outside world, so to speak.”* (Student #11)
		*“I think very few people have really been in a lab like we were […].”* (Student #4)
		*“I would definitely recommend it. […] Especially because, as a regular student [from school], you do not always get the chance, and as far as I know, most internships in those areas are given out based on ‚knowing people’”* (Student #10)
	Career orientation	*Being able to go in and work there gave me a good insight into the job of a biologist or a scientist.”* (Student #12)
		*I personally want to study medicine, so I thought it was cool to see a lab and so on.”* (Student #10)
		*“And that shows you entirely new aspects, especially if you are interested in biology, whether it might be something for your future career. Whether you want to work in a lab or if you can imagine studying biology.”* (Student #6)
		*“You can then start thinking about whether this might be something for you in the future or not.”* (Student #5)

**Table 8:** Selection of quotes from different groups of participants describing ‘Active Science Learning’ as a key success factor.

Theme	Subtheme	Example quotes
Active science learning	Hands-On involvement	*“It’s not a work where you are sitting on the chair the whole time or sitting at the laptop, but it’s also practical, that you are working at the microscope or experimenting or something like that.”* (Student #2)
		*“And at school, we do not really do much pipetting, so I thought it was a pretty cool experience.”* (Student #11)
	Adequate support	*“I found the point that we were practically taken along the best. That it was explained to us in the lab beforehand what we had to do. […] And when we were in these small groups, they also explained what we were currently doing, what was in these solutions and so on, I found that quite good because sometimes I feel that when you do experiments, it’s a bit difficult to understand everything at once and you do the experiment, but in the end, you do not really understand what the evaluation means, I found that good.”* (Student #10)
	Interaction and Integration	*“It was also, well, the thing with the cups, that we were integrated like that, I also found that good.”* (Student #2)
		*“I liked […] that we were so actively involved in the presentations and so on.”* (Student #1)
		*“We did some interactive things, which I thought was good. For example, with the cups […], or when we scanned QR codes and had to vote on something—that was really great.”* (Student #5)
		*“Because it was so interactive, I also found it really exciting to listen.”* (Student #9)
	Visualization	*“That was a kind of completely new first contact because you simply got much closer to it, you could experience and see much more yourself and not just hear about it.”* (Student #1)
		*“I especially liked the second day because we were able to experiment on something very current. I was there and essentially recreated the RNA vaccination, and that was really interesting because it was so relevant, and it helped me understand the Corona vaccination much better. Being able to do that practically myself was really cool.”* (Student #8)
		*“And if you somehow see it yourself or experiment with it or recognize it in a representation or something like that, then that’s of course something good.”* (Student #3)
		*“For me, it was a repetition of what I theoretically did in the internship, or where I watched, so it was great for me to be able to do it myself now.”* (Student #12)
	Autonomy	*“That you have completely different directions open to you regarding what you want to do. And I think you can also come up with what you would like to experiment with yourself.”* (Student #2)
		*“In general, I found it very great that we in the lab did not have this being constantly observed, but that they also let us do it and allowed us to gather our own experiences.”* (Student #12)
		*“I found it interesting that we were able to experiment a lot on our own.”* (Student #7)

**Table 9:** Selection of quotes from different groups of participants describing ‘Inclusion of ethical aspects and discussions’ as a key success factor.

Theme	Subtheme	Example quotes
Inclusion of ethical aspects and discussions	Awareness of diverse perspectives	*“I believe two students commented on this… that they indeed received a more differentiated picture simply and realized how important it is to hear the other side. And that is also a characteristic marker for critical thinking: not only listening to one’s own head.”* (Expert #4, ethics research)
		*“I think especially on the third day one took away new perspectives on how to view situations and one’s stance on them; one could exchange ideas well with others who might have had different opinions; thus, one learned to look at things from another perspective.”* (Student #5)
		*“I thought it was particularly cool during the ethical discussion […] because afterwards we heard other opinions and learned about things we had not even thought about.”* (Student #9)
		*“I liked that we could develop all arguments ourselves and that there were opposing viewpoints on vaccination; these were very new things, too, which changed my perspective completely during the discussion.”* (Student #7)
	Recognition of complexity	*“I saw this during the discussion on mandatory vaccination. The political positions became polarized. Initially, there were three positions, and in the end, there were only two. But all those who expressed themselves said: ‘We see this in a more differentiated way’ in their words. However, even though they see it more differentiated, they have a clearer opinion now. The opinion has taken shape, but not because it was simplified; rather, one first opened up this complex space and then had to reduce it again. And that is already a fantastic outcome.”* (Expert #4, ethics research)
	Personal/Societal relevance	*“So we talked a lot about it, especially on the last day. Now we know which side the [note: vaccine opponents] might represent a bit, and because of that, we could respond to it because we have counterarguments against it, and I think we would actually be quite well equipped.”* (Student #2)
		*“I think it’s also important that topics are discussed which are important for general knowledge, so society can progress.”* (Student #3)
		*“And the discussion at the end of the third day actually has significance for everyone in society.”* (Student #11)
		*“The topic of vaccination affects everyone; whether one should get vaccinated or not affects everyone; thus, during such a project you receive many arguments and various viewpoints explained in a very illustrative way.”* (Student #7)

## 4 Results

The following section outlines the key factors identified from our interview and observational data that fostered students’ interest and engagement in the open schooling vaccination program.

### 4.1 Collaborative planning

Collaborative planning emerged as a key success factor in the development of the educational vaccination program. This approach involved the joint planning and design of educational interventions by all stakeholders, contributing to the overall effectiveness of the program. Experts noted that this method differed significantly from their typical experiences when engaging with the public, such as in school collaborations.

Science experts who participated in the collaborative planning sessions prior to implementation expressed appreciation for the feedback and insights they received from colleagues with diverse areas of expertise. For instance, they valued educational input regarding content difficulty from teachers, who are well-versed in assessing student comprehension.

Collaborative planning also played a crucial role in enhancing the alignment between experts and students, ensuring that the educational intervention was truly student-centered. As highlighted by the teacher during our interview, preparing the experts before their meetings with students, allowed them to gain valuable insights into the students’ existing knowledge of immunobiology. This preparation enabled the experts to tailor their content and approach to match the students’ current understanding, fostering a more meaningful and engaging learning experience.

Moreover, collaborative planning was recognized as essential for fostering long-term cooperation among different experts. Familiarity and shared experiences in developing a cohesive educational concept lowered barriers to future communication and collaboration, making it easier for stakeholders to reconnect and work together again.

### 4.2 Interaction with experts

One of the primary factors contributing to the success of the educational vaccination program was the facilitation of direct interactions with experts. The teacher noted that integrating expert contact into the program allowed for extraordinary learning experiences that transcended typical classroom instruction.

Through these interactions, students gained valuable insights into the professional routines and real-world activities of experts, including hands-on experiences in laboratories. This exposure provided them with a clearer understanding of the practical applications of their studies and the realities of working in science-related fields.

Students also emphasized that the experts were approachable and fostered an environment where mistakes were viewed as part of the learning process. This supportive atmosphere contributed to a low-stress experience, encouraging students to engage more freely without fear of judgment.

Moreover, students appreciated the diversity in age among the experts. Medical students were particularly seen as accessible, creating a welcoming environment for students to ask questions and seek guidance. In contrast, older experts, who were further along in their careers, were perceived as more distant. Data from our observational study confirmed this finding from our interviews, as we noted that students interacted more frequently and intensively with the younger experts.

### 4.3 Exposure to diverse professional environments

Another key factor contributing to the success of the educational vaccination program was the students’ exposure to various workplaces related to vaccination and the underlying science. Both experts and teachers emphasized that learning outside the traditional classroom setting significantly enhanced student engagement.

Through these experiences, students gained valuable insights into the work environments of professionals, as well as the specific activities associated with those settings. The program allowed students to explore concepts they had previously only heard about, providing them with a tangible understanding of what those roles entail.

Students expressed that such exclusive and authentic experiences are not typically available to them, noting that access often depends on pre-existing connections. This realization highlighted the unique opportunity presented by the program, which many students felt was not universally accessible, a sentiment also supported by our observational data, which indicated that students were more engaged and inquisitive in these authentic contexts.

Furthermore, exposure to different work environments proved beneficial for students’ career orientations. Engaging with professionals in real-world settings helped them better understand potential career paths and informed their future decisions.

### 4.4 Active science learning

Active science learning was identified as another key success factor in the educational vaccination program. Students expressed that they enjoyed engaging in practical work a lot, as it made abstract concepts more tangible and relatable. The implementation of hands-on activities allowed students to grasp the experiments they conducted and understand the results they obtained, which seems not always to be given in traditional school experimental settings.

Additionally, students appreciated the integration of interactive formats beyond the laboratory. Elements such as a cup game organized by the medical students and the use of QR codes for live voting during presentations were particularly well-received. These interactive components made listening periods more engaging and fostered greater participation among students, as confirmed by our observational data, which indicated that students were particularly active and enthusiastic during these interactive sessions.

Ultimately, students emphasized the importance of autonomy and making their own decisions in the learning process, particularly in relation to active science learning. They highlighted that engaging in self-directed activities was meaningful. This independence and sense of ownership in completing tasks was a key aspect they appreciated about the program.

### 4.5 Integration of ethical aspects and discussions

The analysis revealed that the inclusion of ethical aspects and discussions was a crucial success factor in the educational vaccination program. Engaging students in a debate about mandatory COVID-19 vaccinations significantly heightened their awareness of diverse perspectives beyond their own. By acknowledging and considering these differing viewpoints, students developed a more nuanced understanding of the issue, enriching their own perspectives in ways they had not previously contemplated.

Our observations indicated that students were particularly engaged during the session on ethical discussions, actively using the information they had collected as a basis for their argumentation. This engagement closely relates to their awareness of other perspectives and the recognition of the complexity that comes with developing a more nuanced understanding.

Ultimately, students highlighted the relevance of discussing contentious topics like vaccination, which impact everyone in society. They noted that through their engagement with various perspectives and the inherent complexities of vaccination discussions, they felt better equipped to confront arguments that diverge from their own viewpoints.

## 5 Discussion

The findings from this study underscore the significant potential of the open schooling approach in effectively addressing complex topics such as vaccination.

Collaborative planning emerged as a crucial success factor in the development of the educational vaccination program. This inclusive process enhanced the overall effectiveness of the program and represented a departure from traditional public engagement practices. Experts valued the feedback and insights shared during collaborative sessions, particularly the contributions from teachers regarding content difficulty. This dialog ensured that educational materials were not only scientifically accurate but also accessible and engaging for students. This is also consistent with the experiences of Borchert and Deisert (30), who describe that multi-professional partnerships between schools and external experts offer numerous learning opportunities for all parties involved.

The Involvement of experts in the planning process fostered a supportive network that is essential for long-term collaboration. The familiarity and shared experiences gained from developing a cohesive educational concept helped to lower barriers to future communication and collaboration. As a result, stakeholders felt more comfortable reconnecting and working together again, which can lead to sustained partnerships in future educational initiatives.

These findings underscore the importance of inclusive planning processes in educational programs, as they not only enhance the quality of the interventions but also build a supportive network among professionals in the field.

The findings also indicate that effective interaction with experts can significantly enhance student interest in science. Although interactions with experts can sometimes be perceived negatively—especially if content is presented in an overly complex manner—thoughtful planning and pre-intervention coaching can yield positive results. Students in this study reported increased interest and engagement, echoing findings from Laursen et al. (31) and Schlopsna and Scheersoi (9), which suggest that direct engagement with scientists can boost students’ attentiveness and enthusiasm. The presence of relatable figures, such as medical students, provided valuable role models, further enhancing students’ connection to the subject matter.

The unique approach of this program—introducing students to multiple science-related settings and diverse professional environments—emerged as a crucial factor in enhancing motivation and engagement. By taking students beyond the confines of the classroom into laboratories and research facilities, the program provided authentic science experiences that are often lacking in traditional educational settings. This approach aligns with Gamse et al. (32), who noted that many educational programs fail to adequately expose students to the varied contexts within the scientific field, underscoring the value of these real-world encounters in fostering sustained interest and enthusiasm for science. Ribeiro, Pinto and Rocha (33) also stated that open schooling approaches in science education provide students with opportunities to engage in authentic, real-world challenges. They highlight the fact that collaborations with experts not only foster meaningful partnerships between schools and universities but also have the potential to enhance students’ scientific attitudes and augment their scientific capital.

Active participation through hands-on activities emerged as another critical factor in fostering interest and engagement in the topic of vaccination. Authentic, hands-on experiences have been shown to significantly enhance student engagement in science education in previous studies (31, 34, 35). The program’s incorporation of game-like activities provided vivid representations of complex concepts, making them more accessible and relatable for students. Participants expressed enthusiasm for this active learning approach, emphasizing how it allowed them to engage directly with scientific processes rather than passively receiving information.

The analysis revealed that incorporating ethical aspects and discussions was another key factor in the success of the educational vaccination program. Collaboration between scientists and ethicists, as recommended by Kabasenche (36), provided students with valuable education in science ethics. Engaging in debates about mandatory COVID-19 vaccinations broadened students’ awareness, encouraging them to consider perspectives beyond their own. By actively exploring these differing viewpoints, students developed a more nuanced understanding of the issue, deepening their perspectives in ways they had not previously considered. This is in line with Reiss (3) who emphazises the multifaceted ethical considerations inherent in vaccination education. He advocates for an approach that transcends purely scientific perspectives, emphasizing the value of examining the topic through various lenses (e.g., by implementation of interdisciplinary science lessons and incorporating pedagogical methods such as discussions, role-playing exercises, and debates).

Students themselves underscored the relevance of discussing contentious topics like vaccination, which have far-reaching implications for society. Through their engagement with various perspectives and the complexities inherent in vaccination discussions, they reported feeling better equipped to confront arguments that diverged from their own views. The preparation of students for the discussions proved to be important in enabling well-founded argumentation and strengthening students’ capacity for informed debate. This aligns with the recommendations of (38), who identifies sufficient time and thorough preparation as central elements of a successful ethical discussion in the classroom.

Through exploring ethical questions and understanding the multifaceted nature of societal issues, students become better prepared to engage in meaningful conversations about public health and policy. This not only fosters their personal growth but also contributes to a more informed and participatory society. In this context, the inclusion of ethical discussions in the vaccination program has proven to be a vital component for developing students’ critical thinking and engagement with societal issues. This approach underscores the importance of integrating ethical aspects into science education, as it promotes a deeper understanding of the complexities surrounding critical public health topics.

## 6 Limitations

This study has several limitations that should be considered when interpreting the findings. First, the study was conducted with a specific group of secondary school students within a single educational program, which limits the generalizability of the results to other contexts or student populations. Additionally, the study’s reliance on qualitative data from interviews and observations provides in-depth insights but may be subject to researcher bias in data interpretation. While a carefully developed coding system was used to analyze the transcribed data, categorizing responses is not entirely free from subjective interpretation. Despite structured coding efforts to reduce bias, the researchers’ perspectives inevitably influence the assignment of statements to categories. To increase transparency in this process, we included multiple original quotes from the interviews in the results section, along with their categorizations and interpretations. This allows readers to critically evaluate the data interpretation and trace the connection between the raw data and the conclusions drawn.

The open schooling program itself was also limited to a three-day timeframe, potentially restricting the extent of long-term impact assessment on student engagement and understanding. Future research with larger, more diverse samples and extended program durations could provide a broader perspective on the effectiveness of open schooling for vaccine education.

## 7 Conclusion

In conclusion, this study highlights the significant value of the open schooling approach as a comprehensive framework for vaccine education, providing students with meaningful engagement in both scientific and societal aspects of vaccination. While open schooling and community-based frameworks have been studied in broader contexts, their integration into formal school systems remains limited. Our findings provide valuable insights into how these approaches can be adapted for effective implementation, particularly for complex and controversial topics like vaccination. By fostering collaboration between schools, local communities, healthcare professionals, and scientists, open schooling enables authentic learning environments where students connect with real-world public health issues, cultivating informed perspectives on vaccination. This model not only deepens students’ understanding of vaccination but also equips them to become active, informed citizens capable of participating in public health discussions and decision-making processes.

Open schooling demonstrates considerable potential to spark and sustain students’ interest in vaccination, combining scientific rigor with social learning. Its focus on real-world contexts, interdisciplinary collaboration, and student-centered pedagogy creates conditions for students to engage deeply with this complex topic. Through hands-on projects and community-based discussions, students analyze diverse perspectives, including ethical, biological, and societal implications of vaccination. The program’s inquiry-driven approach encourages students to question, debate, and build argumentation skills, which not only enriches their grasp of vaccination but also fosters essential competencies in scientific literacy and social discourse.

The findings of this study have significant implications for various stakeholders, including educators, healthcare professionals, and policymakers. The open schooling model not only improves students” understanding of vaccination but also fosters critical thinking and ethical reasoning skills–both crucial for countering vaccine misinformation. For educators, these results underscore the necessity of integrating interdisciplinary, community-based learning into curricula to encourage a more holistic understanding of vaccination, extending beyond traditional classroom methods. Healthcare professionals can draw on these insights to advocate for educational initiatives that promote vaccine literacy among youth, equipping future generations with the knowledge and critical faculties needed to make informed health decisions. Collaborations between schools and healthcare providers could further amplify these efforts. Policymakers are urged to view the open schooling framework as a scalable and effective model for broader educational reforms aimed at improving public health goals. By prioritizing vaccine education within school systems, governments can strengthen community resilience against vaccine hesitancy, fostering a well-informed populace capable of engaging in and supporting public health initiatives.

As educational models continue to adapt to the complexities of the modern world, the open schooling approach offers a promising and adaptable model for teaching critical topics that extend beyond traditional classroom boundaries. Incorporating open schooling principles into educational programs can foster a well-rounded understanding of complex issues like vaccination, empowering students to explore, evaluate, and form nuanced viewpoints that prepare them to engage with the broader societal challenges of the future.

## Data Availability

The raw data supporting the conclusions of this article will be made available by the authors, without undue reservation.

## Footnotes

^1^ https://multipliers-project.org/
